# Calcium: A Nutrient Deserving a Special Issue 

**DOI:** 10.3390/nu2101044

**Published:** 2010-10-05

**Authors:** Susan J. Whiting

**Affiliations:** College of Pharmacy and Nutrition, University of Saskatchewan, 110 Science Place, Saskatoon SK S7N 5C9, Canada; Email: susan.whiting@usask.ca

**Keywords:** calcium, dietary intake, requirement, body weight, adverse effects

## Abstract

Interest in calcium has continued since the 1980s when its role in promoting bone growth and retention was established in clinical trials of children and postmenopausal women. The human nutrition functions now attributed to calcium have expanded beyond bone health to include other conditions such as body weight maintenance. While most efforts have been focused on the findings that dietary intakes are low, there are emerging data on safety concerns of excess amounts. This Special Issue on calcium nutrition, spanning the lifecycle from critically ill neonates through to older adults, has been written by some of the leading researchers in this field.

## 1. Introduction

Interest in calcium has continued since the 1980s when its role in promoting bone growth and retention was established in clinical trials of children and postmenopausal women. These studies helped establish recommended intake levels for calcium in many countries including Canada and the United States [1]. Professional societies, mainly those in osteoporosis [2,3] and related areas [4] have set calcium recommendations as well. Since that time new functions have been proposed for dietary calcium, including a role in preventing body weight gain and/or promoting body weight loss, a theory that has come into its own in the past decade [5,6]. Much interest has focused on calcium intakes; using current recommended cut-offs [1], a high percentage of the population have inadequate intakes, even in countries where dairy consumption is encouraged [7,8]. There has been, however, concern about the safety of high doses [9], and setting an upper intake level (UL) for calcium intake by the Institute of Medicine in 1997 [1] seemed to have lain this to rest. However, recent findings indicating that calcium may be linked to cardiovascular disease [10] have prompted a new examination of this issue. 

Thus the ongoing issues and concerns related to calcium prompted the journal ***Nutrients*** to have a special issue on calcium wherein distinguished calcium researchers were invited to submit recent works or review articles on calcium. The individual articles are now all published and represent a body of work that should be useful for the nutrition community, whether scientists or practitioners, to source up-to-date information on the topics most pertinent to calcium. These papers cover calcium through the lifespan, from infancy [11,12], childhood and adolescence [12,13] and college-age subjects [14] though to older adults [15,16] or include issues relevant to every age group [17,18]. 

## 2. Overview of Topics in Special Issue on Calcium

Topics that are presented are related to ensuring adequate calcium intake for **bone health** [11,12,13,14,15,16], wherein in some of these papers bioavailability is a critical issue [11,12,16]. For infants, the nature of the tube feeding protocol as well as the type of nourishment for critically ill neonates was examined by Rogers *et al.* [11] who found human milk fortified with donor milk-based fortifier was the infant food with the least detrimental effect on calcium losses. In a review on calcium absorption by infants and children, Abrams [12] explains how stable-isotope based studies are the method of choice for measuring calcium absorption. For older adults, this method has been applied to a comparison of calcium supplements for postmenopausal women at risk for osteoporosis [16] to show the higher availability of an algal-based calcium supplement over the widely used calcium carbonate salt. Assessing calcium status in postmenopausal women, along with other bone-related nutrients such as vitamin D, and vitamin K, may be achieved through a food frequency questionnaire [15].

Research on whether calcium plays a role in **body weight maintenance** is included in this issue. Tylavsky *et al.* [13] present new data on the role of calcium intake on body composition in African-American children at risk for overweight/obesity. Soares and She-Ping-Delfos [17] review the literature on postprandial energy metabolism, and provide evidence that higher calcium intakes may affect energy metabolism through excretion of fecal fat. 

**Calcium intakes** are often low. In college-age women, which is a group known to have lower than recommended calcium intakes, Douglas *et al.* [14] examine calcium intakes of students in the United States and in Croatia. Studies of children, adolescents [13] and young adults [13] in this issue provide estimates of calcium intake. In order to readily assess intakes, Pritchard *et al.* [15] provide information on development of a food frequency questionnaire that assesses calcium as well as other bone-related nutrients.

**Safety of calcium** is addressed by Daly and Ebeling [18]. This timely review provides an overview of the health effects of the use of calcium supplements. There may be both risks and benefits of higher levels of supplementation but these authors indicate that moderate calcium intake, that achieved through diet and supplementation when necessary, is the best course of action. 

In conclusion, topics on calcium nutrition, spanning the lifecycle from critically ill neonates through to older adults at risk for osteoporosis and written by some of the leading researchers in fields related to this nutrient, are included in the Special Issue on Calcium. 
